# The effect of preoperative chemotherapy on liver regeneration after portal vein embolization/ligation or liver resection in patients with colorectal liver metastasis: a systematic review protocol

**DOI:** 10.1186/s13643-020-01545-w

**Published:** 2020-12-04

**Authors:** Mihai-Calin Pavel, Raquel Casanova, Laia Estalella, Robert Memba, Erik Llàcer-Millán, Mar Achalandabaso, Elisabet Julià, Justin Geoghegan, Rosa Jorba

**Affiliations:** 1 HPB Unit, Department of General Surgery, Hospital Universitari de Tarragona Joan XXIII, C/ Dr. Mallafrè Guasch, 4, 43005 Tarragona, Spain; 2 Departament de Medicina i Cirugia, Universitat Rovira i Virgili, Reus, Spain; 3 HPB and Liver Transplant Surgery Department, St. Vincent’s University Hospital, Dublin, Ireland

**Keywords:** Liver regeneration, Liver resection, Portal vein embolization, Colorectal cancer liver metastasis, Neoadjuvant chemotherapy, Systematic review

## Abstract

**Introduction:**

Liver resection (LR) in patients with liver metastasis from colorectal cancer remains the only curative treatment. Perioperative chemotherapy improves prognosis of these patients. However, there are concerns regarding the effect of preoperative chemotherapy on liver regeneration, which is a key event in avoiding liver failure after LR. The primary objective of this systematic review is to assess the effect of neoadjuvant chemotherapy on liver regeneration after (LR) or portal vein embolization (PVE) in patients with liver metastasis from colorectal cancer. The secondary objectives are to evaluate the impact of the type of chemotherapy, number of cycles, and time between end of treatment and procedure (LR or PVE) and to investigate whether there is an association between degree of hypertrophy and postoperative liver failure.

**Methods:**

This meta-analysis will include studies reporting liver regeneration rates in patients submitted to LR or PVE. Pubmed, Scopus, Web of Science, Embase, and Cochrane databases will be searched. Only studies comparing neoadjuvant vs no chemotherapy, or comparing chemotherapy characteristics (bevacizumab administration, number of cycles, and time from finishing chemotherapy until intervention), will be included. We will select studies from 1990 to present. Two researchers will individually screen the identified records, according to a list of inclusion and exclusion criteria. Primary outcome will be future liver remnant regeneration rate. Bias of the studies will be evaluated with the ROBINS-I tool, and quality of evidence for all outcomes will be determined with the GRADE system. The data will be registered in a predesigned database. If selected studies are sufficiently homogeneous, we will perform a meta-analysis of reported results. In the event of a substantial heterogeneity, a qualitative systematic review will be performed.

**Discussion:**

The results of this systematic review may help to better identify the patients affected by liver metastasis that could present low regeneration rates after neoadjuvant chemotherapy. These patients are at risk to develop liver failure after extended hepatectomies and therefore are not good candidates for such aggressive procedures.

**Systematic review registration:**

PROSPERO registration number: CRD42020178481  (July 5, 2020).

## Background

### Description of the condition

Colorectal cancer is the fourth most frequently diagnosed cancer and the second cause of death related to cancer [1]. More than 50% of the patients diagnosed with colorectal cancer will develop metastases in the course of their disease [2]. Of these metastases, 20–30% will be confined exclusively in the liver (CRCLM) [3]. To date, liver resection (LR) remains the only curative option for patients with CRCLM [2, 4, 5], with survival rates that may reach 50% and 26% at 5 and 10 years, respectively [6]. The importance of complete treatment of all liver diseases is reflected by the fact that up to 97% of 10-year survivors do not develop recurrence after CRCLM resection [7].

However, more than 80% of CRCLM patients will have unresectable disease at the time of diagnosis [2, 8, 9]. Several prospective trials have shown encouraging results for preoperative chemotherapy, with conversion rates to resectable disease of 12.5–60%, depending on tumor biology and type of regimen used [10–14]. Furthermore, current guidelines recommend preoperative chemotherapy for the majority of CRCLM patients with resectable disease [4, 5]. The justification for this type of recommendations is to lower the probability of microscopic disease, to test the response to the treatment, and to identify the patients with aggressive disease in whom resection would not be indicated [15]. Therefore, the majority of CRCLM patients who reach LR will have received some form of neoadjuvant chemotherapy.

One of the most important complications after LR is liver failure, which leads to a higher probability of major postoperative complications and death [16]. Major post-LR complications are usually associated with a significant increase in hospitalization time and higher postoperative costs [17, 18]. Current data demonstrates a correlation between liver failure and the extent of LR, highlighting the importance of planning a sufficient future liver remnant (FLR) (i.e., the volume of liver to be preserved after LR) when undertaking liver resection [19, 20]. In the context of CRCLM, in order to avoid liver failure, a minimum FLR of 30% is recommended [19, 21, 22]. For smaller FLRs, strategies for manipulating liver volume may be used, such as portal vein embolization (PVE), two-stage hepatectomy, or associating liver partition and portal vein ligation for staged hepatectomy (ALPPS) [19, 22, 23].

Preoperative chemotherapy may cause liver histological changes, such as sinusoidal obstruction syndrome (SOS) and non-alcoholic steatohepatitis (NASH) [21]. SOS has been associated with oxaliplatin regimens, while NASH is described particularly with irinotecan-based chemotherapy [24, 25]. Both syndromes may cause an increase in postoperative complications index, although NASH has especially been associated with higher rates of postoperative liver failure [21, 24].

One of the key events in the liver response to the injury (i.e., LR) is the occurrence of regeneration or hypertrophy. At a cellular level, this process is more accurately described as a compensatory hyperplasia, given that the remaining liver tissue expands in order to meet the organism requirements [26]. In healthy livers, regeneration restores liver volume to more than 80% of the preoperative value, 3 months after major LR [27]. However, several factors may impair adequate hepatic regeneration. The majority of these are also associated with postoperative liver failure: steatosis, fibrosis or cirrhosis, obstructive cholestasis, ischemia, etc. [19, 28]. Since preoperative chemotherapy causes proven histological changes in the liver, a link between neoadjuvant treatment and insufficient hypertrophy of the liver remnant may exist. However, to date, the available data remains controversial. Details about this matter are offered in the subchapter “How the intervention might work?”.

### Description of the intervention

Current US and European guidelines establish the preferred neoadjuvant chemotherapy depending on the liver disease characteristics [4, 5]. However, there is a great degree of variability on the indications for neoadjuvant chemotherapy and on the type of treatment used, depending on each hospital’s local protocol. For this reason, definitive conclusions concerning the oncological results as well as the occurrence of postoperative complications related to the chemotherapy are difficult to obtain outside randomized control trials or systematic reviews. The effect of neoadjuvant chemotherapy on postoperative liver regeneration is subject to the same variability and remains uncertain.

### How the intervention might work

The effect of neoadjuvant chemotherapy on liver regeneration may be considered from several different perspectives.

First, neoadjuvant chemotherapy can cause histological changes in the liver which may impair regeneration after surgery or post-embolization. As shown by several studies, steatosis and NASH are related to preoperative chemotherapy [21, 24, 25]. Liver steatosis reduces hypertrophy after major hepatectomy in animal models [29]. In addition, some publications have shown lower regeneration rates after major hepatectomy in obese patients [30]. Furthermore, there are studies that have demonstrated a direct correlation between lobular inflammation or fibrosis and liver regeneration rate [31]. Other authors mention the association of SOS with lower liver regeneration rates and increased indicators of liver failure [32]. However, the deleterious effect of neoadjuvant chemotherapy on liver regeneration are probably less evident in patients submitted to minor hepatectomies, since in these cases the percentage change in future liver volume is lower than after major hepatectomy [33].

Second, the impact of the number of preoperative chemotherapy cycles on liver regeneration is still not known. Some studies describe lower regeneration rates with more than six cycles of treatment [34], while other studies did not find such differences in the post-procedure hypertrophy rates [31].

Third, the time interval between completion of chemotherapy and the procedure could be important. Some studies report differences in the post-procedure regeneration rates in patients with less than 8 weeks of chemotherapy-free interval, especially when dealing with bevacizumab regimens [35]. However, other studies have failed to reproduce the same results [31].

Finally, the type of chemotherapy might be important. This debate is generally related to regimens containing bevacizumab. This molecule is a monoclonal antibody that targets vascular endothelial growth factor (VEGF) [36]. Its addition to classical chemotherapy regimens has showed an improved response and prolonged disease-free survival rates in patients with initially unresectable disease [37]. Concerns about the possible deleterious effect of bevacizumab on liver regeneration have been raised after experimental studies have shown that neutralization of VEGF inhibited proliferative activity of hepatocytes [38]. However, this effect in human patients is still debated [35].

This systematic review will study the effect of neoadjuvant chemotherapy and of its characteristics (type, number of cycles, and time from the end of treatment until intervention) on liver regeneration.

In accordance with current guidelines, our systematic review protocol was registered with the International Prospective Register of Systematic Reviews (PROSPERO) on July 5, 2020 (registration number CRD42020178481).

## Objectives

The main objective is to assess the effects of neoadjuvant chemotherapy on liver regeneration after LR or PVE in patients with CRCLM when compared to patients without chemotherapy before the procedure (defined as LR or PVE).

Secondary objectives are as follows:

- Evaluate the impact of type of chemotherapy, number of cycles, and time between end of treatment and procedure on liver regeneration after LR or PVE in patients with CRCLM
- Assess the association between liver hypertrophy rate (defined below in the “Outcomes”) section and index of postoperative liver failure/liver dysfunction in patients with neoadjuvant chemotherapy.

## Methods

### Study eligibility criteria

Studies selection will be performed according to the PICO (Population, Intervention, Comparison and Outcomes) criteria of Preferred Reporting Items for Systematic Reviews and Meta-Analyses (PRISMA) described below [39] and detailed in Table 1: Preferred Reporting Items for Systematic Reviews and Meta-Analyses Protocols (PRISMA-P) checklist.

**Table 1:** PRISMA-P 2015 Checklist

Section/topic	No.	Checklist item	Information reported		Line number(s)
			Yes	No	
**Administrative information**					
**Title**					
Identification	1a	Identify the report as a protocol of a systematic review	☒	☐	4
Update	1b	If the protocol is for an update of a previous systematic review, identify as such	☐	☒	
**Registration**	2	If registered, provide the name of the registry (e.g., PROSPERO) and registration number in the Abstract	☒	☐	54
**Authors**					
Contact	3a	Provide name, institutional affiliation, and e-mail address of all protocol authors; provide physical mailing address of corresponding author	☒	☐	6, 19
Contributions	3b	Describe contributions of protocol authors and identify the guarantor of the review	☒	☐	488
**Amendments**	4	If the protocol represents an amendment of a previously completed or published protocol, identify as such and list changes; otherwise, state plan for documenting important protocol amendments	☒	☐	420
**Support**					
Sources	5a	Indicate sources of financial or other support for the review	☒	☐	484
Sponsor	5b	Provide name for the review funder and/or sponsor	☐	☒	No sponsor
Role of sponsor/funder	5c	Describe roles of funder(s), sponsor(s), and/or institution(s), if any, in developing the protocol	☐	☒	No sponsor
**Introduction**					
**Rationale**	6	Describe the rationale for the review in the context of what is already known	☒	☐	61
**Objectives**	7	Provide an explicit statement of the question(s) the review will address with reference to participants, interventions, comparators, and outcomes (PICO)	☒	☐	152
**Methods**					
**Eligibility criteria**	8	Specify the study characteristics (e.g., PICO, study design, setting, time frame) and report characteristics (e.g., years considered, language, publication status) to be used as criteria for eligibility for the review	☒	☐	166
**Information sources**	9	Describe all intended information sources (e.g., electronic databases, contact with study authors, trial registers, or other grey literature sources) with planned dates of coverage	☒	☐	242
**Search strategy**	10	Present draft of search strategy to be used for at least one electronic database, including planned limits, such that it could be repeated	☒	☐	253, 683
***Study records***					
Data management	11a	Describe the mechanism(s) that will be used to manage records and data throughout the review	☒	☐	269
Selection process	11b	State the process that will be used for selecting studies (e.g., two independent reviewers) through each phase of the review (i.e., screening, eligibility, and inclusion in meta-analysis)	☒	☐	277
Data collection process	11c	Describe planned method of extracting data from reports (e.g., piloting forms, done independently, in duplicate), any processes for obtaining and confirming data from investigators	☒	☐	289
**Data items**	12	List and define all variables for which data will be sought (e.g., PICO items, funding sources), any pre-planned data assumptions and simplifications	☒	☐	303
**Outcomes and prioritization**	13	List and define all outcomes for which data will be sought, including prioritization of main and additional outcomes, with rationale	☒	☐	314
**Risk of bias in individual studies**	14	Describe anticipated methods for assessing risk of bias of individual studies, including whether this will be done at the outcome or study level, or both; state how this information will be used in data synthesis	☒	☐	335
***Data***					
**Synthesis**	15a	Describe criteria under which study data will be quantitatively synthesized	☒	☐	355
	15b	If data are appropriate for quantitative synthesis, describe planned summary measures, methods of handling data, and methods of combining data from studies, including any planned exploration of consistency (e.g., *I* ^2^, Kendall’s tau)	☒	☐	360, 390
	15c	Describe any proposed additional analyses (e.g., sensitivity or subgroup analyses, meta-regression)	☒	☐	409
	15d	If quantitative synthesis is not appropriate, describe the type of summary planned	☒	☐	368
**Meta-bias(es)**	16	Specify any planned assessment of meta-bias(es) (e.g., publication bias across studies, selective reporting within studies)	☒	☐	337, 418
**Confidence in cumulative evidence**	17	Describe how the strength of the body of evidence will be assessed (e.g., GRADE)	☒	☐	348

This checklist has been adapted for use with systematic review protocol submissions to BIOMED Central Journals from Table 3 In Moher D et al.: Preferred Reporting Items for Systematic Review and Meta-Analysis Protocols (PRISMA-P) 2015 Statement. Systematic Reviews 2015 4:1

An editorial from the editors-in-chief of systematic reviews details why this checklist was adapted—Moher D, Stewart L & Shekelle P: Implementing PRISMA-P: Recommendations for prospective authors. Systematic Reviews 2016 5:15

#### Types of studies

Randomized controlled trials (RCTs), including cluster RCTs, controlled (non-randomized) clinical trials (CCTs) or cluster trials, controlled before-after (CBA) studies, prospective and retrospective comparative cohort studies, and case-control or nested case-control studies will be included. Cluster randomized, cluster non-randomized, or CBA studies will be included only if there are at least two intervention sites and two control sites. We will include studies independently of geographic location or year of publication. We will accept unpublished material and abstracts from congresses. There will be no language restrictions. Cross-sectional studies, case series, case reports, systematic reviews, meta-analysis, and experimental studies on animals will be excluded. Editorials, letters, or commentaries will be excluded during the screening of titles and abstracts.

#### Type of participants

We will include adult patients with CRCLM and with indication to perform LR or PVE, irrespective of the number of lesions or their localization. At least two volumetric estimations of FLR, one before and one after the procedure (LR or PVE) will be required for the inclusion of the study in the current review. The usual indication to perform PVE is an insufficient FLR. Accordingly, a separate analysis for LR and PVE indications will be performed.

#### Type of interventions

In order to achieve the primary objective, the type of intervention to be taken into account will be neoadjuvant chemotherapy, irrespective of type, number of cycles, or other characteristics. According to the National Cancer Institute, neoadjuvant chemotherapy is defined as “treatment given as a first step to shrink a tumor before the main treatment, which is usually surgery” [40].

In order to achieve the secondary objectives, we will perform sub-analysis for the following type of interventions:

1. Type of chemotherapy: addition of bevacizumab to the chemotherapy regimen.
2. Number of cycles: the intervention will be administration of more than 6 cycles of neoadjuvant chemotherapy. Depending on the data found in the selected studies, this number may be changed.
3. Time between the end of chemotherapy and the procedure (LR or PVE): the intervention will be considered when this wait is less than 8 weeks. Depending on the analysis performed in the selected studies, this number may be changed.

#### Comparators

For the primary objective, the control group will include patients without chemotherapy submitted to the procedure (defined as LR or PVE). We will accept studies in which the control group contains patients with benign disease or other types of neoplasia. However, these studies will be carefully analyzed to detect possible biases of selection.

For the secondary objectives, the control group can include patients with chemotherapy that do not meet the type of intervention mentioned above, patients without chemotherapy (similar to primary objective), or both.

#### Outcomes

##### Primary outcomes

1. *Liver regeneration/hypertrophy rate*: *future liver remnant regeneration rate (FLR*^*3*^*)*, calculated as follows:
  $$ \mathrm{FL}{\mathrm{R}}^3=\frac{{\mathrm{FLR}}_f-{\mathrm{FLR}}_i}{{\mathrm{FLR}}_f} $$

where FLR_*f*_ is the final future liver remnant (after the procedure—LR or PVE) and FLR_*i*_ is the initial future liver remnant (before the procedure). The timing of FLR_*f*_ will depend on the data reported in the selected studies, but we expect mainly volumetric data in the first month after the procedure, giving that most part of the hypertrophy occurs in this interval.

2. *Surrogate volumetric data*:
  - Total liver volume changes
  - Changes in the volume of the liver to be resected (derived from pre- and post-procedure volumes in the case of PVE and pre-procedure volumes and weight of the resected liver in the case of LR)

##### Secondary outcomes

1. *Postoperative liver failure or liver dysfunction.* Accepted definition of liver failure will be:
  - 50–50 criteria as proposed by Balzan: association of a prothrombin time of less than 50% and serum bilirubin of more than 50 μmol/L on 5th postoperative day [16]
  - Bilirubin peak of more than 120 μmol/L during the postoperative period of a major hepatectomy [41]
  - Grade B and C of post-hepatectomy liver failure according to International Study Group of Liver Surgery [42].

### Information sources

#### Electronic searches

Literature search strategies will be conducted using medical subject headings (MeSH) and text words related to the objectives of this systematic review. We will search the following electronic databases:

1. PubMed (1990 to present)
2. Scopus (1990 to present)
3. Web of Science (1990 to present)
4. Embase (1990 to present)
5. Cochrane Central Register of Controlled Trials (1996 to present)

The key words used to perform the search in each electronic database are as follows: (regeneration OR hypertrophy) AND chemotherapy AND (liver OR hepatic) AND (metastasis OR metastases OR metastatic OR secondary). The details of the search are shown in the Appendix.

Before proceeding to write the manuscript draft, the search of the literature will be updated, in order to identify any new publication which could be relevant to the objectives of this systematic review.

#### Searching other resources

Reference lists of all primary studies and review articles will be manually searched for additional references. Authors of published studies that were selected for this review will be contacted if needed in order to ask them to identify other published and unpublished studies. Errata or retractions from eligible studies will be searched on PubMed, and the date this was done will be reported in the review.

### Data collection and analysis

#### Data management

Literature search will be loaded in a specially created Mendeley folder in order to access to titles and abstracts and will be listed in a specially created Excel file with the following coding: included, not included, and 2nd look. Several methods will be used to identify duplicate publications, according to Cochrane Handbook for Systematic Reviews of Interventions: trial identification numbers, juxtaposing author names, location and setting of the study, comparing sample sizes, specific details of the interventions, date and duration of the study, outcomes, and text of the abstract [43].

#### Selection process

Two review authors (MP and RC) will independently screen titles and abstracts of all the potential studies identified as a result of the search, and code them as “included”, “not included,” or “2nd look”. Full text of study reports coded as “included” or “2nd look” will be retrieved, and two review authors (MP and RC) will independently analyze the full text, identifying studies for inclusion and recording reasons for exclusion of the ineligible studies. Study authors will be contacted when additional information will be needed to resolve questions about eligibility. Any disagreement between the two authors will be solved by a third reviewer (RJ). Duplicates and collate multiple reports of the same study will be identified and excluded; thus, each study rather than each report will be the unit of interest in the review. The selection process will be recorded in sufficient detail to complete a PRISMA flow diagram and a table of excluded studies features.

#### Data collection process

An Excel-based data collection form will be used to record study characteristics and outcome data as described below in subchapters “Data Items” and “Outcomes and Prioritization.” The data collection will be piloted on five studies previously identified as significant for this review. Two review authors (MP and RC) will independently extract study characteristics and outcome data from included studies. Any disagreement between the two authors will be solved by a third reviewer (RJ). To ensure consistency across reviewers, calibration exercises will be conducted before starting the review. Corresponding authors of selected studies will be contacted to resolve any uncertainties (three e-mail attempts at maximum). In order to extract data not reported in a numeric format, graphically presented data will be translated into usable format using OriginPro v 7.5 from OriginLab. Before the final revision of the draft, another search will be performed in order to identify duplicate publications among the selected articles, following the same previously described methodology.

#### Data items

The following study characteristics and outcomes will be extracted:

1. *Methods*: study design, total duration of study and run-in period, number of study centers and location, study setting, withdrawals, and date of study
2. *Participants*: number, mean age, age range, gender, inclusion and exclusion criteria
3. *Interventions*: intervention, comparison, and any co-interventions
4. *Outcomes*: primary and secondary outcomes specified and collected, and time points reported
5. *Notes*: funding for trial and notable conflicts of interest of trial authors

#### Outcomes and prioritization

The main outcome of this systematic review will be FLR^3^, which represents the hypertrophy of the remnant liver after surgery. This outcome is derived from the estimated volume of the remnant before and after the intervention (LR or PVE). Even though both interventions induce liver hypertrophy, presumably FLR^3^ after LR or PVE will not be comparable. Therefore, FLR^3^ will be analyzed separately after each one of the interventions. FLR^3^ data will be expressed as mean ± standard deviation (SD). If data is offered in other forms (median–range or median–interquartile range (IQR)), mean ± SD will be calculated following the recommendations of Cochrane Handbook for Systematic Reviews of Interventions [43], whenever possible.

Regarding the timing of post-procedure volume calculation, homogenous data are expected in PVE studies. The majority of preoperative PVE protocols plan surgery 4 weeks after the embolization procedure. However, the timing of remnant volumetry after LR could vary between studies. The majority of included studies most likely perform volume calculation 1 month after the procedure, when 80% of liver hypertrophy has occurred. Whenever remnant volume has been calculated at several time points, the one obtained at 1 month after LR will be chosen. The time of post-procedure volumetry will be registered for each study.

The secondary outcome will be liver failure/dysfunction as defined above. This outcome will be calculated only for patients submitted to LR. This outcome will try to determine whether there is a correlation between a possible lower hypertrophy rate in patients with neoadjuvant chemotherapy and subsequent liver dysfunction rate.

### Assessment of bias

Two investigators (MP and RC) will independently assess risk of bias for the included studies. Risk of bias will be assessed by the Risk Of Bias in Non-randomized Studies of Interventions (ROBINS-I) tool for non-RCT studies [44]. In this tool, risk of bias is assessed within specified domains, including (1) bias due to confounding, (2) bias in selection of participants into the study, (3) in classification of interventions, (4) bias due to deviations from intended interventions (5) bias due to missing data, (6) bias in measurement of outcomes, (7) bias in selection of the reported result, and (8) overall bias. Since assessments are inherently subjective and there are no strict and objective criteria to judge bias within the ROBINS-I tool, disagreements will be resolved via discussion between the two investigators or by the intervention of a third (RJ). If any RCT meeting the inclusion criteria are found, the evaluation of bias will be performed according to the Cochrane risk-of-bias tool for randomized trials (RoB 2) [45].

### Quality assessment for all outcomes

The quality of evidence for all outcomes will be determined with the Grading of Recommendations Assessment, Development and Evaluation (GRADE) system [46]. Quality will be evaluated as high, moderate, low, or very low. The evaluation of quality will be independently performed by two of the authors (MP and RJ).

### Data synthesis

If selected studies are sufficiently homogeneous in design and comparators, we will perform a meta-analysis of reported results.

#### Measures of treatment effect

Dichotomous data (liver failure or dysfunction as previously defined or presence of ascites, encephalopathy) will be analyzed by using risk ratio (RR) with 95% confidence interval (CI) and continuous outcomes (FLR^3^ or surrogate volumetric data—total liver volume changes and changes in the liver volume to be resected) as mean difference or standardized mean difference when different scales are used (e.g., FLR^3^ vs surrogate volumetric data).

If a study is suspected of comprising skewed data, this is commonly indicated by reporting medians and interquartile ranges. When this is found, transformations to mean differences will be carried out. If this is not possible due to lack of data, the data will be considered as skewed [47]. If the data are skewed, a meta-analysis will be not performed, though a narrative summary will be provided instead.

#### Unit of analysis issues

The unit of analysis will be individual participants affected by liver metastasis and candidates for LR or PVE. If any cluster randomized studies are unexpectedly found, the data will be included in the analysis if the results are adjusted for intra-cluster correlation. If any cross-over randomized studies are found, the data prior to the cross-over will be included. When a study has more than two treatment groups, the additional treatment arms will be presented. Where the additional treatment arms are not relevant, they will not be taken into account.

#### Dealing with missing data

Investigators or study sponsors will be contacted to verify key study characteristics and obtain missing numerical outcome data (e.g., when a study is presented as abstract only). If this information is not available from the study authors, it will be obtained, where feasible, by using calculations provided in the Cochrane Handbook for Systematic Review of Interventions [43]. The impact of including such studies will be assessed in a sensitivity analysis. If we are unable to calculate the standard deviation from standard error, interquartile range, or *P* values, we will impute standard deviation as the highest standard deviation in the remaining studies included in the outcome.

#### Assessment of heterogeneity

Clinical heterogeneity will be tested by considering the variability in participant factors among trials (e.g., age) and trial factors (randomization concealment, blinding of outcome assessment, losses to follow-up, treatment type, co-interventions). Statistical heterogeneity will be tested using the chi-squared test (significance level: 0.1) and *I*^2^ statistic (0 to 40%: might not be important; 30 to 60%: may represent moderate heterogeneity; 50 to 90%: may represent substantial heterogeneity; 75 to 100%: considerable heterogeneity). If high levels of heterogeneity among the trials exist (*I*^2^ > =50% or *P* < 0.1), the study design and characteristics in the included studies will be analyzed. The source of heterogeneity by subgroup analysis or sensitivity analysis will be explained.

#### Data synthesis

Each outcome will be calculated using the statistical software RevMan 5.1, according to the current version of the Cochrane Handbook for Systematic Reviews of Interventions [43]. The Mantel-Haenszel method will be used for the fixed effect model if tests of heterogeneity are not significant. If statistical heterogeneity is observed (*I*^2^ > =50% or *P* < 0.1), the random effects model will be chosen. Data will be presented in text and tables, in order to summarize the characteristics and findings of included studies. The analysis will describe the findings and associations within individual studies as well as among all the studies included in this review.

#### Subgroup analysis and investigation of heterogeneity

Subgroup analysis will be used to investigate possible sources of heterogeneity, based on the following parameters:

1. General characteristics of included patients (age, sex)
2. Timing of post-procedure volumetry
3. Type of procedure (hepatectomy vs PVE)

Sensitivity analysis will be performed to explain the source of heterogeneity:

1. Analysis of the material retrieved (full text vs abstract only, preliminary data vs final results, published vs unpublished material)
2. Risk of bias (performing analysis by omitting studies evaluated as of high risk of bias)

### Amendments

If there is a need to amend this protocol, the date of each amendment will be registered, describing the change and giving the rationale in this section. Changes will not be incorporated into the protocol.

### Reaching conclusions

Conclusions will be based on findings from the quantitative or narrative analysis of the studies included in this review. We will avoid making recommendations for clinical practice but we will focus on the remaining uncertainties in the field and the need for future clinical investigation.

## Discussion

To date, there is sufficient data to conclude that postoperative liver regeneration is a key factor in avoiding postoperative liver failure. Factors associated with postoperative liver failure are also associated with impaired liver regeneration [19, 28]. Furthermore, predicted insufficient FLR volume represents an indication to perform alternative techniques in order to stimulate liver hypertrophy such as preoperative PVE, two-stage hepatectomy, or ALPPS [19, 48]. Since liver failure is associated with higher rates of postoperative death, it is important to evaluate whether neoadjuvant chemotherapy may cause a deficit in liver regeneration.

However, there are no systematic reviews that analyze specifically the association between chemotherapy characteristics and post-procedure hypertrophy. A number of important prospective randomized controlled trials establish as a primary objective the oncological results or the postoperative complication rates [3, 37, 49, 50]. Furthermore, the heterogeneity of regimens used, the different protocols of treatments, and the lack of volumetric data in published studies makes it difficult to draw definitive conclusions regarding the role of neoadjuvant chemotherapy in liver regeneration without a properly conducted systematic analysis. Therefore, the need of a systematic review centered on this issue is evident.

## Data Availability

Data sharing is not applicable to this article, as no datasets were generated or analyzed during the current study. Authors’ information The HBP unit of Hospital Universitari de Tarragona Joan XXIII is an emergent group, formed by several surgeons with an important background in HBP surgery. Our unit is the only one in Tarragona province approved to perform liver resections for colorectal liver metastasis. MCP is currently the coordinator of the HBP Committee of Tarragona province, Spain. Together with LE, he is responsible for the management of the waiting list of patients with CRCLM for the University Hospital of Tarragona Joan XXIII. RJ is the chief of General Surgery Department of University Hospital of Tarragona Joan XXIII. RM is the coordinator of the HBP unit.

## Appendix

### Search strategy for electronic databases

#### Pubmed

(“Liver”[Mesh] OR “Liver”[tiab] OR “Liver Neoplasms”[Mesh] OR “Hepatic”[tiab] OR “Hepatectomy”[tiab])

AND

(“Neoplasm metastasis”[Mesh] OR metasta*[tiab] OR “secondary”[tiab])

AND

(“Liver Regeneration”[Mesh] OR “Regeneration”[Mesh] OR “Regeneration”[tiab] OR “Hypertrophy”[Mesh] OR “hypertrophy”[tiab])

AND

((“chemotherapy”[tiab] OR “Antineoplastic Agents”[Mesh]) AND (“preoperative”[tiab] OR “before”[tiab]))

Resultats →50 referències [ accés: pubmed.pdf ]

#### Scopus

TITLE-ABS((“Liver” AND “Hepatic” OR “Hepatectomy”) AND (metasta* OR “secondary”) AND (“Regeneration” OR “hypertrophy”) AND “chemotherapy” AND (“preoperative” OR “before”))

Resultats → 62 referències [ accés: scopus.pdf ]

#### Web of Science

TS = ((“Liver” AND “Hepatic” OR “Hepatectomy”) AND (metasta* OR “secondary”) AND (“Regeneration” OR “hypertrophy”) AND “chemotherapy” AND (“preoperative” OR “before”))

*Período de tiempo: Todos los años. Bases de datos: WOS, CCC, DIIDW, KJD, MEDLINE, RSCI, SCIELO.*

*Idioma de búsqueda = Auto*

Resultats →193 referències [accés: webofscience.pdf ]

#### Embase

(‘liver’/exp OR ‘liver’:ti,ab) AND (‘liver neoplasms’/exp OR ‘hepatic’:ti,ab OR ‘hepatectomy’:ti,ab OR ‘liver resection’/exp) AND (‘metastasis’/exp OR metasta*:ti,ab OR ‘secondary’:ti,ab) AND (‘liver regeneration’/exp OR ‘regeneration’/exp OR ‘regeneration’:ti,ab OR ‘hypertrophy’/exp OR ‘hypertrophy’:ti,ab) AND (‘chemotherapy’:ti,ab OR ‘chemotherapy’/exp OR ‘antineoplastic agents’/exp) AND (‘preoperative’:ti,ab OR ‘preoperative period’/exp OR ‘before’:ti,ab)

Resultats → 341 referències [accés: embase.pdf]

#### Cochrane

((“Liver” AND “Hepatic” OR “Hepatectomy”) AND (metasta* OR “secondary”) AND (“Regeneration” OR “hypertrophy”) AND “chemotherapy” AND (“preoperative” OR “before”)) in Title Abstract Keyword - (Word variations have been searched)

Resultats →7 referències [accés: cochrane.txt ]

## Abbreviations

ALPPS: Associating liver partition and portal vein ligation for staged hepatectomy
CBA: Controlled before-after study
CCT: Controlled clinical trial
CRCLM: Colorectal cancer liver metastases
FLR: Future liver remnant
FLR^3^: Future liver remnant regeneration rate
GRADE: Grading of Recommendations, Assessment, Development and Evaluation
LR: Liver resection
MeSH: Medical Subject Headings
NASH: Non-alcoholic steatohepatitis
PICO: Population, Intervention, Comparison and Outcomes
PRISMA: Preferred Reporting Items for Systematic Reviews and Meta-Analyses
PRISMA-P: Preferred Reporting Items for Systematic Reviews and Meta-Analyses Protocols
PROSPERO: International prospective register of systematic reviews
PVE: Portal vein embolization
RCT: Randomized controlled trial
RoB 2: Cochrane risk-of-bias tool for randomized trials
ROBINS-I: Risk of Bias in Non-randomized Studies of Interventions
SOS: Sinusoidal obstruction syndrome
VEGF: Vascular endothelial growth factor
